# Information Gathering Over Time by Breast Cancer Patients

**DOI:** 10.2196/jmir.5.3.e15

**Published:** 2003-08-27

**Authors:** Melisa J Satterlund, Kevin D McCaul, Ann K Sandgren

**Affiliations:** ^1^North Dakota State UniversityDepartment of PsychologyFargo NDUSA; ^2^Roger Maris Cancer CenterFargo NDUSA

**Keywords:** Breast cancer, Internet, Internet use, Internet search

## Abstract

**Background:**

Unlike many patients of the past, today's health-care users want to become more informed about their illnesses, and they want the most current information. The Internet has become a popular way to access current information, and since its introduction more people are turning to it to find medical information. Studies report that anywhere from 36% to 55% of the American population that use the Internet is using the Internet to research medical information, and these percentages have been rising. Cancer is 1 of the top 2 diseases about which people seek information on the Internet. Some studies have specifically asked whether breast cancer patients access the Internet for medical information; estimates range from 10% to 43% of breast cancer patients who use the Internet, with higher usage being associated with more education, greater income, and younger age.

**Objective:**

To identify where breast cancer patients find medical information about their illness and to track changes over time, from active treatment to survivorship status.

**Methods:**

Participants were 224 women who had been recently diagnosed with Stage I, Stage II, or Stage III breast cancer. Each woman was contacted approximately 8 months and 16 months after diagnosis and was asked about 10 different information sources they could have used to obtain information or support about their breast cancer.

**Results:**

Eight months after diagnosis, the top 3 information sources used by women were books (64%), the Internet (49%), and videos (41%). However, at follow-up (16 months after diagnosis), the most frequently cited information source was the Internet (40%), followed by books (33%), and the American Cancer Society (17%). We found that women continued to use the Internet as a means of gathering information even after their treatment ended. Significant unique predictors of Internet use were more years of formal education and younger ages. Cancer stage was not a significant predictor of Internet use.

**Conclusions:**

Previous research has been mixed about the percentage of cancer patients who use the Internet to gather information about their illnesses. The results of the present study corroborate 2 other data sets of breast cancer patients, as just over 44% of the women reported using the Internet after diagnosis. Sixteen months after diagnosis, the percentage of women using the Internet dropped slightly, but other chief sources dropped sharply at that time. The Internet continues to play an important role for cancer survivors after medical treatment has ended, and health professionals can use this knowledge to provide their patients with Internet advice.

## Introduction

Patients of the 21st century are not like patients of the past — many want to become more informed about their illness, and they want the most current information [1- 5]. The increased desire to acquire information has been accompanied by dramatic increases in the proportion of people in the population who have Internet access. Thus, we are starting to see a shift in how patients obtain medical information [6]. In the past, consumers sought information from health professionals, books, media (eg, videos), and support networks (eg, the American Cancer Society). Now the information source of first choice may be the Internet.

Internet access has continually increased since the Internet was introduced. In 2002, 169 million people in the United States had current access, an increase of 10% over the previous year [7]. When people were asked why they use the Internet, the most-important reason was to quickly obtain information [8]. The Internet offers several advantages in addition to rapid information acquisition: finding information online is relatively easy, people can share their experiences with others, and they can research anything in privacy. However, finding information online has its drawbacks. Some people are still unable to access the Internet easily, and finding reliable, credible sources may be difficult [5,9- 11]. In a recent review of studies evaluating the quality of Web sites, Eysenbach et al [12] reported that 70% of the studies concluded that quality is a problem on the Web, and only 9% of the studies evaluated the quality of sites positively. These data suggest that searchers are typically unlikely to find reliable and credible sources. However, Fogel et al [13] found that breast cancer patients chose as their favorite Web sites those containing reliable and credible information.

Some studies have examined whether people are using the Internet to obtain medical information. Lebo reported that 36% of Internet users accessed medical information on the Web [8]. A similar estimate was obtained from a representative sample of the US population, in which 40% of the respondents with Internet access said they looked for health care advice or information [14]. However, Baker et al [14] also reported that the Internet had little effect on health care utilization, as indexed by the number of physician visits or telephone contacts. In 2000, the Pew Internet & American Life Project reported that 55% of Americans with access to the Internet used it for medical purposes [11]. In that study, individuals who used the Internet for medical information, identified as "health seekers," were reinterviewed to obtain more detailed data. More women (63%) than men (46%) consulted the Web for health information. Approximately 30% of the health seekers reported using the Internet to seek advice about health about once a month, and 29% reported using the Internet about once a week. Less-healthy individuals reported greater weekly use (32%) than individuals in excellent health (23%). Additional findings were that Internet users liked the idea that they could access medical information any time of the day and could do so anonymously.

Cancer is 1 of the top 2 diseases about which people seek information on the Internet, with approximately 35% of Americans using the Internet to gather information about cancer [15]. Several studies have been conducted to determine whether and how cancer patients use the Internet to research their disease. Mills and Davidson asked cancer patients (colorectal, lung, breast, prostate, gynecological, or gastric) where they obtained information and found that fewer than 10% reported using the Internet [16]. The main source of information used by patients was the hospital consultant, followed by the general practitioner. Diefenbach et al [6] examined the explanations that men diagnosed with prostate cancer gave for their for treatment decisions, finding that only 7% of the patients reported using the Internet to make their decisions. Similarly, Raupach et al [17] found that fewer than 7% of women diagnosed with breast cancer used the Internet as a means of gathering information about their cancer.

Pereira et al asked a similar question of breast cancer patients and reported much higher Internet use [5]. Nearly half (43%) of the women said they used the Internet to look for information related to their cancer. Of those who used the Internet, over 90% used it to find more information about their cancer and its treatment. Breast cancer Internet users were younger and more educated than nonusers. Fogel et al also asked whether breast cancer patients used the Internet as an information-gathering source, and reported results similar to those of Pereira et al [3,5]. Fogel et al found that 42% of the women used the Internet for medical information, and that Internet users tended to be younger with a higher education level [3]. Internet users had higher incomes and were more likely to be white. No differences were found for the stage of breast cancer.

Given the rapid expansion of Internet use, the number of cancer patients who use the resource, and how they do so, is likely to change rapidly. It is important to assess where patients are seeking information about their disease and to track changes over time. The data in the present study come from a clinical trial of telephone therapy for newly-diagnosed cancer patients [18]. We provide data about 2 important comparisons that add to prior research. First, we asked patients to describe their use of many different information sources, so we could compare Internet use to other possible ways of gathering information. Second, we followed patients over time, from active treatment to survivorship status. The longitudinal design allowed for a characterization of how breast cancer patients obtain information during different phases of their disease.

## Methods

### Participants

We report data obtained in the context of a clinical trial testing 2 interventions to help women cope with breast cancer. The results of the intervention study are reported elsewhere [18]. Participants, recruited from 2 regional cancer treatment centers, were 224 women who had been recently diagnosed with Stage I (n = 110), Stage II (n = 85), or Stage III (n = 29) breast cancer. Women with Stage 0 or Stage IV diagnoses were excluded from the study. Sixty-nine women (22.5% of those asked) declined to participate, with the most common reason being "not interested." Because initial analyses showed no treatment differences between conditions on the types of information gathered at either interval, the data presented are collapsed across conditions. Most of the women were married (77%) and Caucasian (96%), and they ranged in ages from 30 to 84 (mean = 54.5).

### Procedure

Approximately 9 weeks (mean = 9.0) after diagnosis, women were recruited to take part in the current study. After consent was obtained, we conducted baseline telephone interviews. Eight months after diagnosis (mean = 8.4), women were reinterviewed and asked about their information-gathering behaviors since they had been diagnosed with breast cancer. At nearly 16 months after diagnosis (mean = 15.6), the women were contacted a final time and asked whether or not they had used any of the same information sources since the last time we had talked to them. All data collection took place between August 1999 and September 2002.

We began the study with 237 participants. The 224 participants described above were retained at the first follow-up period (95% of the original 237) and the data from 217 participants (92% of the original 237) were available at the second follow-up period. The small number of dropouts would have little impact on the overall Internet use statistics presented here. On 2 background variables, however, the dropouts at the 16-month interval did differ slightly from those participants who stayed in the study until the end. Specifically, dropouts were more likely to have a higher stage of cancer (means = 1.90 and 1.59, *t*_235_= 1.89, *P*= .06) and a lower income (means = 2.15 and 2.78, *t*_228_= 2.02, *P*= .05). As reported below, neither of these 2 variables predicted Internet use in the logistic regression equation at the 16-month interval.

### Measures

Participants reported whether they used 10 different sources to obtain information or support about their breast cancer. Three of the information sources were followed by an open-ended question to allow the participant to expand on "yes" answers. Participants were asked whether they had:

1. read any books about breast cancer (and, if yes, to provide the title)
2. taken part in a support group
3. participated in "I Can Cope," an educational program sponsored by the American Cancer Society (ACS) that provides support to breast cancer survivors
4. met a "Reach to Recovery" volunteer, another program sponsored by the American Cancer Society, in which persons diagnosed with breast cancer can talk with a trained volunteer about their cancer
5. participated in the "Look Good, Feel Good" program, an American Cancer Society program that teaches female cancer patients beauty techniques to reduce appearance-related side effects of cancer and cancer treatments
6. watched any videos (and, if yes, to provide the title)
7. called the National Cancer Institute Information Service
8. contacted the American Cancer Society
9. contacted the Y-Me National Hotline, a 24-hour hotline in which trained breast cancer survivors answer questions and provide support to women who have questions about breast cancer
  and/or
10. used the Internet to gather any information (and, if yes, the topics that you researched).

When contacted for the second interview, women were not asked about the "I Can Cope" program, because it did not generate enough responses at the first interview.

## Results

### Data Analyses

We conducted 3 kinds of analyses. First, descriptive statistics were used to describe Internet use data. Second, individual chi-square analyses were used to test differences in Internet use over time. Third, tests of association between background variables (eg, age) and Internet use were conducted in 2 ways: (1) using individual chi-square tests or point-biserial correlations, and (2) using logistic regression to test the unique contributions of the background variables to Internet use.

Table 1 shows the percentages of women who said that they used each of the 10 information sources. After diagnosis, the top 3 sources used by the women were books, the Internet, and videos. The most frequently cited book read by the women was "Dr. Susan Love's Breast Book" [19,20]. Infrequently used sources were the Y-Me National Hotline and the "I Can Cope" program. At follow-up (16 months), the most frequently cited information source was the Internet, followed by books, and the American Cancer Society (see Table 1). Women continued to use the Internet as a major means of gathering information even after their treatment ended. The other top cited sources declined dramatically over that time, a significant drop for both books, *χ ^2^*_1_= 32.43, *P*< .001 and videos, *χ ^2^*_1_= 8.32, *P*= .004.

**Table 1:** Information sources used by patients 8 months and 16 months after diagnosis

**Information Source**	**Women Who Said They Used Information Source(%)**	
	**8 Months(n = 224)**	**16 Months(n = 217)**
Books	64	33
Internet	49	40
Videos*	41	13
Reach a Recovery Volunteer*	39	11
American Cancer Society	25	17
Look Good Feel Good	14	12
Support groups	9	10
National Cancer Information Service	9	8
Y-Me National Hotline	4	1
I Can Cope	2	NA

^*^ Significant changes were observed between the periods after diagnosis and at follow-up, *χ ^2^ P*< .01.

Table 2 shows the types of information women were seeking while using the Internet. These data were generated in response to an open-ended question the interviewers asked when patients said that they used the Internet to gather information (ie, "Can you tell me the topics that you researched?"). Six general topics appeared most frequently in patients' responses to the open-ended questions. Eight months after diagnosis, the 2 topics mentioned by the most women were treatment information and specific breast cancer information. At follow-up (16 months), the 2 topics mentioned by the most women were specific breast cancer information and medications.

**Table 2:** Most common topics of information sought on the internet 8 months and 16 months after diagnosis

**Topic**	**Women who said they sought information on the topic(%)**	
	**8 Months(n = 110)**	**16 Months(n = 86)**
Treatment information	26	14
Specific breast cancer information	26	21
Medications	23	20
Medical institutions/resources	15	5
General cancer information	14	14
Tamoxifen	13	9

Given the rapid expansion of the Internet, one might expect that Internet use would differ from when our first participants were assessed (1999) to when our last participants were assessed (2002). We tested this possibility by dividing our sample into 4 approximately-equal groups, differing by an earlier vs later diagnosis date. No significant differences in Internet usage were observed for these groups at either measurement interval, although the trend was for greater use by women who were diagnosed most recently (eg, at the 8-month follow-up, the percentage using the Internet was 46% during the earliest diagnosis period and 56% during the latest period), *χ ^2^*_3_= 1.74, *P*= .42.

### Predictors of Internet Use

We tested several predictors of Internet use during the interval after diagnosis. Cancer stage was not a significant predictor, *χ ^2^*_2_= 1.74, *P*= .42. However, more years of formal education, higher income levels, and younger age were significantly related to greater Internet use after diagnosis ( *r*= 0.28, *r*= 0.18, and *r*= -0.36 respectively; for all three, *P < .01*). At follow-up, cancer stage again failed to predict Internet use, *χ ^2^*_2_= .12, *P*= .94. More years of formal education, higher income levels, and younger age all remained significantly associated with greater internet use ( *r*= 0.26, *r*= 0.20, and *r*= -0.25 respectively; for all 3, *P*< .01).

To assess the relative importance of the predictors of Internet use, we conducted logistic regression analyses for both time intervals, entering cancer stage, education, income, and age simultaneously. Table 3 presents the results of those analyses. The data are similar for both intervals and differ in only one way from the reported individual associations. Similar to the individual reports, cancer stage was unrelated to Internet use, but younger age and more years of education were significantly related to use at the 8-month and 16-month intervals. However, unlike the individual associations, income was no longer a significant predictor of use in the logistic regressions. This result may be at least partly attributed to the shared variance between years of education and income ( *r*= 0.25); once education entered the regression equation, income no longer predicted unique variance in Internet use.

**Table 3:** Summary of logistic regression analyses predicting Internet use at both follow-up intervals

**Variable**	**Odds Ratio**	**95% Confidence Interval**	***P***
8-month interval			
Cancer stage	0.93	0.60-1.42	.72
Age	0.94	0.92-0.97	<<.001
Education	1.25	1.08-1.44	.002
Income	1.09	0.87-1.37	.47
16-month interval			
Cancer stage	0.88	0.57-1.36	.57
Age	0.97	0.94-0.99	.016
Education	1.21	1.06-1.39	.006
Income	1.19	0.93-1.52	.17

## Discussion

Previous research has been mixed about the percentage of cancer patients who use the Internet to gather information about their illness. Mills and Davidson [16] reported that fewer than 10% of cancer patients use the Internet, but Fogel et al and Pereira et al found that 43% of breast cancer patients use the Internet [3,5]. The results of the present study corroborate the latter findings, as just over 44% of the women reported using the Internet. The percentage of women using the Internet after diagnosis was 49%, declining slightly to 40% at follow-up. In contrast, the use of videos dropped sharply — 68% at follow-up. Similarly, the use of books dropped by 48%. It is important to know that the Internet continues to play an important role for cancer survivors after medical treatment has ended, a finding that is best identified using the sort of longitudinal design employed here.

Reported Internet use was measured only from retrospective recall of patients involved in our study, which is a methodological limitation. However, several findings were consistent with other investigations, providing some confidence in the data collection method and in the possibility that we can generalize from this study to other people and places. As just noted, for example, the overall level of reported Internet use was nearly identical to levels reported in 2 recent studies of breast cancer patients: just less than half of patients say they use the Internet. In addition, correlational data fit with earlier findings, in that Internet use was higher among better educated, younger, and wealthier women. These data probably reflect ease of access and perhaps confidence in using the Internet as an information source.

Although the reliance on self-report may not detract much from the study findings, other limitations should be noted. Because we used open-ended questioning, the data concerning exactly what women learned from the Internet are sketchy. They appeared to search for specific treatment information (eg, data concerning Tamoxifen, treatment regimens) as well as general cancer information. However, to obtain more-precise information about Internet searching, it would be preferable to collect diary data and to report the specific sites that patients use to obtain information. The use of a diary would also help solve another limitation of the present study — reliance on long-term recall. Women recalled activities from several months earlier in describing their Internet searching, and we know that a better data collection strategy would avoid depending on such long-term memories. Finally, we did not ask about one very important source of information: health professionals, especially physicians. It would have been good to know about patients' perceptions of whether they obtained their most-important information from their own health-care providers.

Despite the study limitations, the present findings have implications for future research and practice. Follow-up research could explore the role that health providers play in Internet use. Do physicians encourage Internet use? Do patients who are using the Internet have different kinds of interactions with their health-care team? Do some patients rely more on the Internet for information than on what they learn from their own health-care team? These sorts of questions are likely to become increasingly relevant as more patients turn to the Internet for health information. But the questions are already important, given that nearly half of patients appear to be using the Internet and because, according to our results, over time the Internet becomes the most-frequently used information source. The latter finding also points to the need to investigate the exact sites that patients are using to obtain information. Are they sites that contain accurate information? How do patients explore the Internet to find accurate and useful information?

Efforts to evaluate cancer information on the Internet have already begun [12]. Biermann et al [1] conducted a systematic evaluation of Web sites identified when searching for the topic of "Ewing's Sarcoma" using 4 search engines. The searches often generated irrelevant Web sites and dead ends, and many patients spent numerous hours searching but were unable to find specific and reliable information they needed. In a different study, researchers provided Internet training sessions to cancer patients and their family members about how to access specific information related to their cancer [2]. All the patients found the sessions to be helpful, and they were interested in participating in additional sessions. Given the value that many patients appear to be finding in the Internet as an information source, it is incumbent on health professionals to explore ways to facilitate best use of the resource to ensure that patients are obtaining quality information.

## References

[1] Biermann J S, Golladay G J, Greenfield M L, Baker L H (1999). Evaluation of cancer information on the Internet. Cancer.

[2] Edgar Linda, Greenberg Arlene, Remmer Jean (2002). Providing internet lessons to oncology patients and family members: a shared project. Psychooncology.

[3] Fogel Joshua, Albert Steven M, Schnabel Freya, Ditkoff Beth Ann, Neugut Alfred I (2002). Use of the Internet by women with breast cancer. J Med Internet Res.

[4] Leydon G M, Boulton M, Moynihan C, Jones A, Mossman J, Boudioni M, Mcpherson K (2000). Cancer patients' information needs and information seeking behaviour: in depth interview study. BMJ.

[5] Pereira JL, Koski S, Hanson J, Bruera ED, Mackey JR (2000). Internet usage among women with breast cancer: an exploratory study. Clin Breast Cancer.

[6] Diefenbach Michael A, Dorsey Jenevie, Uzzo Robert G, Hanks Gerald E, Greenberg Richard E, Horwitz Eric, Newton Fredrick, Engstrom Paul F (2002). Decision-making strategies for patients with localized prostate cancer. Semin Urol Oncol.

[7] NetRatings, Inc (2003). Nielsen//NetRatings. Global internet population grows an average of four percent year-over-year.

[8] Lebo H (2003). The UCLA Internet report: surveying the digital future. Year three. Los Angeles, CA.

[9] Brodie M, Flournoy R E, Altman D E, Blendon R J, Benson J M, Rosenbaum M D (2000). Health information, the Internet, and the digital divide. Health Aff (Millwood).

[10] Eng T R, Maxfield A, Patrick K, Deering M J, Ratzan S C, Gustafson D H (1998). Access to health information and support: a public highway or a private road?. JAMA.

[11] Fox Susannah (2000). The online health care revolution: How the web helps Americans take better care of themselves.

[12] Eysenbach Gunther, Powell John, Kuss Oliver, Sa Eun-Ryoung (2002). Empirical studies assessing the quality of health information for consumers on the world wide web: a systematic review. JAMA.

[13] Fogel J, Albert S M, Schnabel F, Ditkoff B A, Neugut A I (2001). Quality of health information on the Internet. JAMA.

[14] Baker Laurence, Wagner Todd H, Singer Sara, Bundorf M Kate (2003). Use of the Internet and e-mail for health care information: results from a national survey. JAMA.

[15] Larkin M (2000). Online support groups gaining credibility. Lancet.

[16] Mills Moyra E, Davidson Robin (2002). Cancer patients' sources of information: use and quality issues. Psychooncology.

[17] Raupach Jane C A, Hiller Janet E (2002). Information and support for women following the primary treatment of breast cancer. Health Expect.

[18] Sandgren Ann K, Mccaul Kevin D (2003). Short-term effects of telephone therapy for breast cancer patients. Health Psychol.

[19] Love Susan M, Lindsey Karen (1995). Dr. Susan Love's Breast Book.

[20] Love Susan M, Lindsey Karen (2000). Dr. Susan Love's Breast Book.

